# Co-application of a biosolids product and biochar to two coarse-textured pasture soils influenced microbial N cycling genes and potential for N leaching

**DOI:** 10.1038/s41598-020-78843-9

**Published:** 2021-01-13

**Authors:** Sanjutha Shanmugam, Sasha N. Jenkins, Bede S. Mickan, Noraini Md Jaafar, Falko Mathes, Zakaria M. Solaiman, Lynette K. Abbott

**Affiliations:** 1 UWA School of Agriculture and Environment (M079), The University of Western Australia, 35 Stirling Highway, Perth, WA 6009 Australia; 2 UWA Institute of Agriculture (M082), The University of Western Australia, 35 Stirling Highway, Perth, WA 6009 Australia; 3 Department of Land Management, Faculty of Agriculture, Universiti Putra Malaysia, 43400 Serdang, Selangor Malaysia; 4 Bioscience Pty Ltd, 488 Nicholson Road, Forrestdale, Perth, WA 6112 Australia

**Keywords:** Environmental impact, Carbon cycle, Agroecology, Microbial ecology

## Abstract

Co-application of biochar and biosolids to soil has potential to mitigate N leaching due to physical and chemical properties of biochar. Changes in N cycling pathways in soil induced by co-application of biological amendments could further mitigate N loss, but this is largely unexplored. The aim of this study was to determine whether co-application of a biochar and a modified biosolids product to three pasture soils differing in texture could alter the relative abundance of N cycling genes in soil sown with subterranean clover. The biosolids product contained lime and clay and increased subterranean clover shoot biomass in parallel with increases in soil pH and soil nitrate. Its co-application with biochar similarly increased plant growth and soil pH with a marked reduction in nitrate in two coarse textured soils but not in a clayey soil. While application of the biosolids product altered in silico predicted N cycling functional genes, there was no additional change when applied to soil in combination with biochar. This supports the conclusion that co-application of the biochar and biosolids product used here has potential to mitigate loss of N in coarse textured soils due to N adsoption by the biochar and independently of microbial N pathways.

## Introduction

Addition of treated industrial wastes such as biosolids to soil is an established practice for improving soil fertility^1–3^ and increasing microbial diversity, structure and function^4–7^. Biosolids may contain high concentrations of plant macro- and micro-nutrients in addition to chemical contaminants and pathogens^8–12^. Furthermore, application of biosolids to soil can result in excessive nutrient leaching^13,14^ (especially on degraded sandy soils) and this can lead to eutrophication of nearby aquatic systems and fish kills^15^. Nutrient leaching can be particularly problematic on sandy, coarse-textured soils in south-western Australia^16^. Therefore, one way to reduce the potential for N leaching in these soils is to apply biosolids in combination with other soil conditioners such as biochars, clay and straw^9,17–22^.

Co-application of clay minerals with sewage sludge has been shown to influence N retention in sewage sludge^22^. The biosolids product used in this study is a modified form of high quality biosolids that has been used as a slow release fertiliser for sandy soils in south-western Australia. The biosolid product is formulated with clay and lime^20^ and therefore called Lime amended Biosolid with Clay (LaBC). In an incubation study, the clay and lime components of LaBC ameliorated both soil acidity and water repellence of sandy soils^20^. However, in the same study, LaBC increased microbial biomass and net-N mineralisation over a 30-week period, potentially increasing the risk of nitrate leaching^20^. Thus, further research is required to underpin the additional effects of LaBC on the soil microbial community, including those related to soil N mineralisation and nutrient retention, due to the presence of clay^23^ and lime^24^. Hence, assessment of N release from LaBC, its availability for plant growth, and potential for N leaching are important criteria^25^ for assessment of LaBC as a soil amendment.

Co-application of LaBC and biochar has potential to reduce N accumulation in soil and leaching from the biosolid component in coarse-textured soils based on a number of studies demonstrating interactions between biochars, clay and N-cycling from biosolids^17–22^. For example, addition of biochar to a soil amended with biosolids reduced nitrate leaching over periods up to 5 months in two silt-loam soils collected from pasture sites^17^. However, mechanisms responsible for inhibition of nutrient leaching by the biochar-biosolid combination were not identified. Some biochars have potential to improve soil fertility and other agronomically related benefits by altering nutrient cycles, especially the nitrogen (N) cycle^26^. Their capacity to adsorb nitrogenous compounds due to polar and non-polar sites on their surfaces has been linked to increasing nutrient retention in soil and could explain the reduced nitrate availability in soils receiving co-applications of biochar and biosolids^14,27,28^. In some cases, biochars can increase soil cation and anion exchange capacities as well as soil pH^29^ leading to improved N retention^27,30^. Co-application of biosolids and biochar will also lead to interactions with the soil microbial community and alter activities including N cycling. To our knowledge, only one study to date has investigated potential mechanisms underlying the impact of co-application of biosolids and biochar. Wang et al.^31^ demonstrated changes in microbial community structure but did not explore changes to bacterial mediated nitrification and denitrification processes as a possible biological mechanism for reducing soil nitrate concentration following amendment with biosolid combined with biochar^32,33^. While there is a possibility that N retention caused by co-application of biosolids and biochar could be due to the physical/chemical properties of the biochar itself (i.e. polar/non-polar surfaces, cation/anion exchange capacity), an additional mechanism could involve changes to N cycling pathways, especially nitrification and denitrification in the soil.

Biochars have been reported to both increase and decrease soil microbial biomass and/or activity^18,26^ and this can alter nitrification and denitrification leading to a reduction in soil nitrate concentration^32,33^. In a coarse-textured soil, decreased microbial biomass C (MBC) following application of organic/inorganic N fertilisers combined with increasing rates of a woody Eucalyptus biochar was associated with decreased soil N mineralisation and organic matter decomposition^18^. In another experiment, the same biochar decreased nitrate leaching in a coarse-textured soil^19^. Biochars are very diverse^34^, and while some can influence soil microbial N cycling and activities when applied alone^32,33,35^ or in combination with manure^36^, little is known about the microbial interactions following co-applications of biochar and biosolids. Nevertheless, interactions between biochar and biosolids could be beneficial in sandy soils, particularly in south-western Australian pasture soils by altering N cycling and reducing N leaching. Thus, this study focussed on investigating the impact of co-application of LaBC and a biochar (Table 1) on N cycling gene dynamics and biological fertility in three soils differing in texture.

**Table 1:** Basic chemical properties of soil amendments (LaBC and biochar).

Characteristics	Biochar^b^ (Simcoa Ltd.)	LaBC^a^ (Water Corporation, WA)
Total C content (%)	73.8 (± 6.89)	3.1 (± 0.89)
Total N content (%)	0.4 (± 0.05)	0.3 (± 0.03)
Total inorganic N (mg kg^−1^)	4.2 (± 0.41)	183.1 (± 11.43)
Organic matter (%)	–	6 (± 0.05)
Moisture content (%)	–	27 (± 1.08)
Lime (%)	–	3 (± 0.01)
Clay (%)	–	64 (± 4.21)
EC (mS cm^−1^)	0.54 (± 0.09)	4.0 (± 0.04)
pH (CaCl_2_)	7.6 (± 0.91)	8.2 (± 0.65)

Values represent the means (and standard errors) of three replicates (n = 3).

^a^Data adapted from Shanmugam et al.^20^.

^b^Data adapted from Mickan et al.^49^.

In our experiment, LaBC was applied either alone or with a wood-based biochar in three soils with different textures, fertility and management history to assess responses in soil biological processes in relation to growth of subterranean clover after 8 weeks. The three soils (Soils 1, 2 and 3) were a sand, a sandy loam and a clayey soil respectively. The aim was to determine whether there was an interaction between LaBC and biochar on N cycling and growth of subterranean clover. It was expected that N released from LaBC would be adsorbed by the biochar to different extents in each soil^17,21,37^ and that the extent of any interactions would depend on soil texture, fertility and agricultural management^38,39^. Soil microbial biomass and microbial diversity and function were expected to respond to a more favourable microhabitat provided by the biochar^26,40–43^ in combination with slow release of N from the biosolids in LaBC.

A novel aspect of this research was the investigation of microbial processes underpinning interactions between the biosolids product LaBC and the wood-based biochar to predict conditions under which beneficial interactions might occur. We hypothesised that (i) LaBC would be a useful soil amendment for subterranean clover and that addition of biochar would further increase benefits in two coarse-textured soils but not in a clayey soil, (ii) co-application of LaBC with biochar would reduce soil nitrate concentration and the potential for leaching in the coarse textured soils, (iii) based on its clay, lime and N components, LaBC would increase the relative abundance of N-cycling genes in coarse-textured soils but not in a clayey soil without any additional influence of the biochar, and (iv) soil microbial biomass would be higher when LaBC and biochar were applied together than when applied separately, especially in the coarse-textured soils.

## Results

### Plant growth

**Figure 1:**
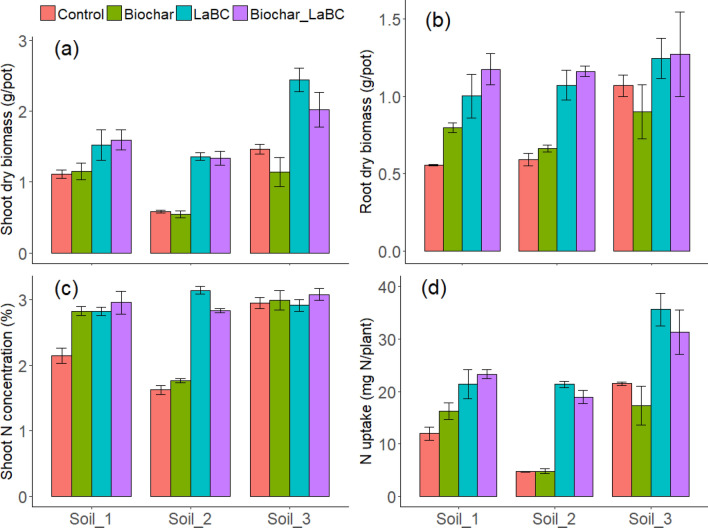
The influence of LaBC, Biochar and Biochar + LaBC amendments on (**a**) shoot dry biomass, (**b**) root dry biomass, (**c**) shoot N concentration and (**d**) plant N uptake for subterranean clover grown on three soils of contrasting textures (Soils 1, 2 and 3; sand, sandy loam and clay, respectively) after 8 weeks. Error bars represent standard errors (n = 3).

Shoot biomass was significantly increased following soil amendment with LaBC and with co-application of LaBC and biochar after 8 weeks in all three soils (Fig. 1a). Application of LaBC alone increased shoot biomass in Soils 2 and 3 by 134% and 67% respectively (P < 0.05). Co-application of LaBC and biochar reduced shoot biomass (P < 0.05; 29%) compared to application of LaBC alone. Root biomass was significantly influenced by some treatments (Fig. 1b). There was a significant increase in root biomass with application of LaBC in Soils 1 and 2 (P < 0.05) but not in Soil 3 (P > 0.05). When biochar was co-applied with LaBC, there was no significant effect on root biomass compared with LaBC applied alone in each soil (P > 0.05).

Shoot N concentration and content were both significantly influenced by soil amendment in the two coarse-textured soils (P < 0.05; Fig. 1c). In Soil 1, all soil amendments increased shoot N concentration (P < 0.05). In Soil 2, application of LaBC alone and in combination with biochar increased shoot N concentration (P < 0.05). In Soil 3, no significant effect occurred. Plant N uptake increased with application of LaBC when applied alone and in combination with biochar in all three soils, but this was more pronounced in Soils 2 and 3 (P < 0.05; Fig. 1d).

### Soil chemical analyses

There was a significant increase in the pH of all soils with application of LaBC alone and in combination with biochar (P < 0.05), but biochar applied alone did not alter soil pH (P > 0.05; Table 2). There was a significant increase in soil nitrate concentration in all soils with application of LaBC (P > 0.05; Table 2), but biochar had no effect on soil nitrate concentration in any soil when applied alone (P > 0.05; Table 2). In Soil 1, LaBC applied alone or in combination with biochar significantly decreased soil ammonium concentration (P < 0.05; Table 2) but there was no difference between treatments with LaBC with or without biochar (P > 0.05; Table 2). In contrast, in Soils 2 and 3, there was a marked increase in soil ammonium concentration with application of LaBC (P < 0.05), but application of biochar with LaBC reduced the magnitude of the effect to that of biochar alone (Table 2). There was little influence of either LaBC or biochar when applied alone on soil microbial biomass, but both soil microbial biomass N (MBN) and soil microbial biomass C (MBC) increased (P < 0.05) with co-application of LaBC and biochar (Table 2).

**Table 2:** Effect of soil amendments (Biochar, LaBC, Biochar + LaBC) on the soil physico-chemical parameters of three soils after 8 weeks in the presence of subterranean clover.

Soil	Treatments	MBC (µg g soil^−1^)	MBN (µg g soil^−1^)	Soil TN (µg g soil^−1^)	Nitrate–N (µg g soil^−1^)	Ammonium–N (µg g soil^−1^)	pH (CaCl_2_)
Soil 1 (sand)	Control	107.49 ± 23.08^b^	17.21 ± 3.7^b^	1.52 ± 0.43^c^	0.33 ± 0.2^c^	1.19 ± 0.25^a^	4.5 ± 0.02^b^
	Biochar	139.55 ± 46.83^ab^	22.35 ± 7.5^ab^	2.02 ± 0.11^bc^	1.31 ± 0.16^c^	0.71 ± 0.13^ab^	4.4 ± 0.01^b^
	LaBC	225.72 ± 45.15^ab^	36.15 ± 7.23^ab^	7.02 ± 0.55^a^	6.45 ± 0.5^a^	0.56 ± 0.05^b^	6.1 ± 0.01^a^
	Biochar + LaBC	279.84 ± 47.15^a^	44.82 ± 7.55^a^	3.9 ± 0.99^b^	3.42 ± 0.98^b^	0.48 ± 0.08^b^	6.3 ± 0.01^a^
Soil 2 (sandy loam)	Control	145.65 ± 42.65^ab^	23.32 ± 6.83^ab^	0.52 ± 0.23^b^	0.26 ± 0.26^c^	0.27 ± 0.14^c^	4.2 ± 0.01^b^
	Biochar	94.02 ± 16.2^b^	15.06 ± 2.59^b^	0.73 ± 0.26^b^	0.34 ± 0.28^c^	0.39 ± 0.03^bc^	4.3 ± 0.01^b^
	LaBC	285.21 ± 49.87^a^	45.68 ± 7.99^a^	13.06 ± 3.2^a^	12.07 ± 3.3^a^	0.99 ± 0.15^a^	6.6 ± 0.04^a^
	Biochar + LaBC	245.94 ± 80.81^ab^	39.39 ± 12.94^ab^	5.26 ± 0.64^a^	4.54 ± 0.61^b^	0.72 ± 0.10^ab^	6.6 ± 0.02^a^
Soil 3 (clay)	Control	822.47 ± 291.56^ns^	131.72 ± 46.69^ns^	0.94 ± 0.21^b^	0.72 ± 0.23^b^	0.23 ± 0.06^b^	5.3 ± 0.05^c^
	Biochar	585.24 ± 24.63^ns^	93.73 ± 3.95^ns^	1.61 ± 0.48^b^	1.23 ± 0.59^b^	0.38 ± 0.12^b^	5.2 ± 0.01^c^
	LaBC	962.86 ± 198.05^ns^	154.2 ± 31.72^ns^	4.56 ± 0.9^a^	3.76 ± 1.05^a^	0.79 ± 0.15^a^	5.8 ± 0.07^a^
	Biochar + LaBC	639.6 ± 45.77^ns^	102.43 ± 7.33^ns^	4.6 ± 0.92^a^	4.07 ± 0.9^a^	0.53 ± 0.03^ab^	5.5 ± 0.18^b^

MBC (microbial biomass C); MBN (microbial biomass N); TN (total N).

Values presented are means ± standard error of the mean, *n* = 3; letters in superscript show treatments that differ significantly.

### Soil bacterial community structure

**Figure 2:**
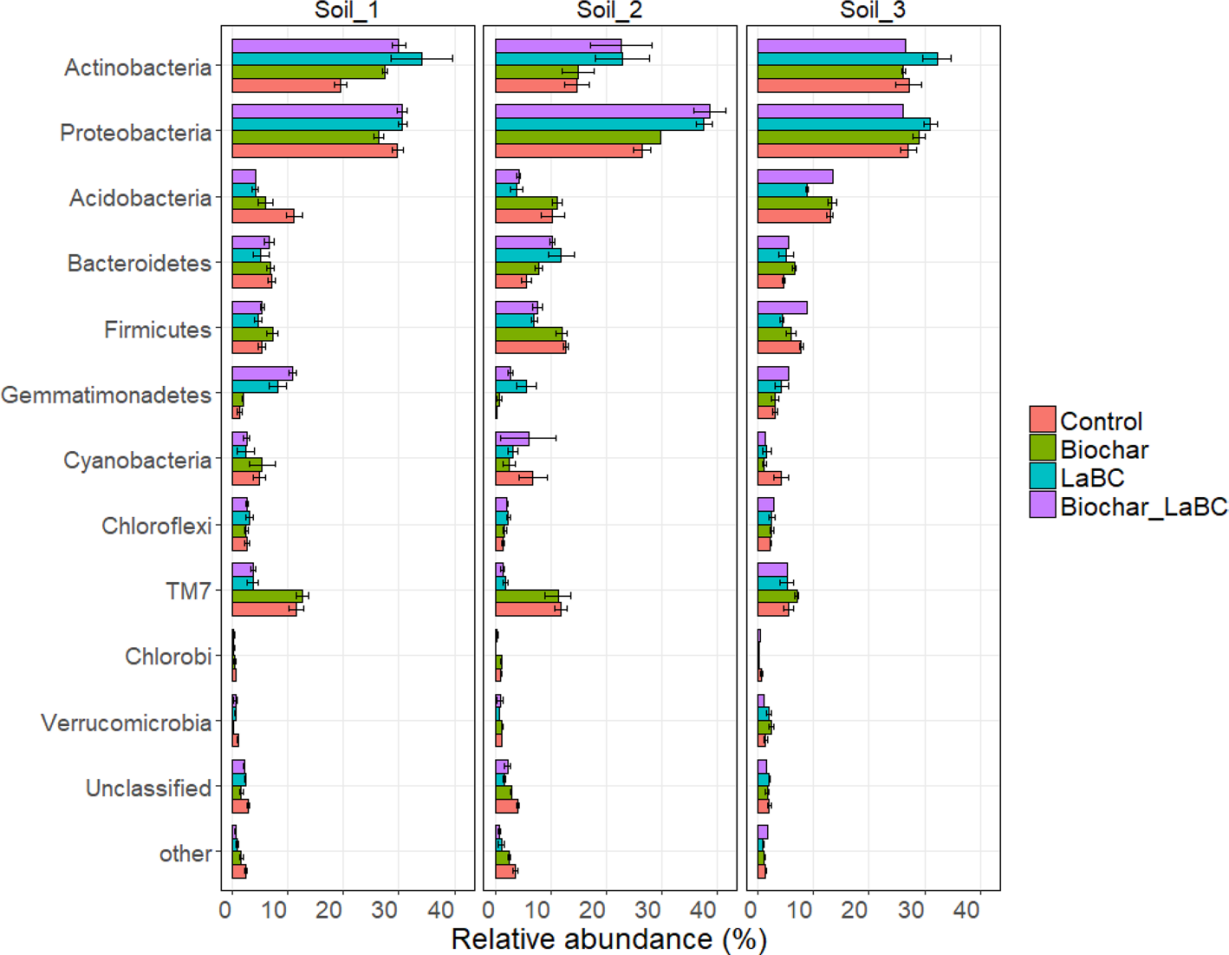
Relative abundance of bacterial phyla for three soils of contrasting textures (Soils 1, 2 and 3; sand, sandy loam and clay respectively) amended with LaBC, Biochar and Biochar + LaBC or unamended (control) after 8 weeks with subterranean clover. Error bars represent standard errors (n = 3).

The bacterial community composition was significantly influenced by soil amendments for all soils (Fig. 2). Proteobacteria and Actinobacteria were the dominant bacterial phyla, jointly accounting for 40–60% of the total OTUs. For Soil 1, there was a significant increase in the relative abundance of Actinobacteria with all amendments (Fig. 2). There was also a marked increase in the relative abundance of Gemmatimonadetes when LaBC was applied alone or with biochar. In contrast, the relative abundance of the Acidobacteria decreased with application of all amendments. There was also a marked decrease in the relative abundance of TM7 when LaBC was applied alone and in combination with biochar. For Soil 2, similar effects of treatments were observed for Actinobacteria, Proteobacteria, Gemmatimonadetes, Acidobacteria and TM7 to those in Soil 1 (Fig. 2) except for a significant decrease in the relative abundance of Actinobacteria compared to Proteobacteria and a decrease in the relative abundance of Bacteriodetes when LaBC was applied alone or in combination with biochar. For Soil 3, the response of the soil bacterial community to amendments was more variable than for the other two soils (Fig. 2). Addition of LaBC alone significantly decreased the relative abundance of Firmicutes and Acidobacteria and increased the relative abundance of Actinobacteria.

### Relationship between bacterial community structure, soil amendment and soil type

**Figure 3:**
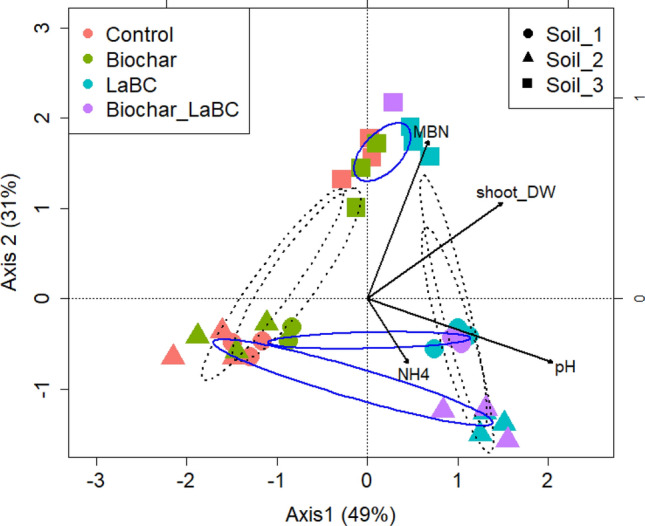
Canonical correspondence analysis (CCA) biplot showing the relationship between soil type, soil amendments and measured soil variables for three soils of contrasting textures (Soils 1, 2 and 3; sand, sandy loam and clay respectively) amended with LaBC, Biochar and Biochar + LaBC after 8 weeks with subterranean clover. Arrows represent the measured variables (pH, NH_4_^+^, MBN). Blue ellipses separate the samples by soil types whilst black dash line ellipses separate the samples by treatment.

The relationships between key environmental variables (pH, NH_4_^+^, MBN), bacterial community structure, dominance of phyla, amendments and soil types were investigated using CCA analysis (Fig. 3). The bacterial communities in Soil 3 responded least to soil amendments. Soils 1 and 2 clustered according to treatment, with LaBC applied alone or in combination with biochar separating from the unamended control and biochar-only treatments along axis 1 with increasing pH. The LaBC and LaBC + Biochar treatments were associated with an increase in ammonium concentration and relative abundance of Bacteroidetes in Soil 2 whereas they were associated with an increase in MBN and the relative abundance of Chloroflexi in Soil 1.

### Changes in abundance of N and C cycling genes in response to soil amendments

**Figure 4:**
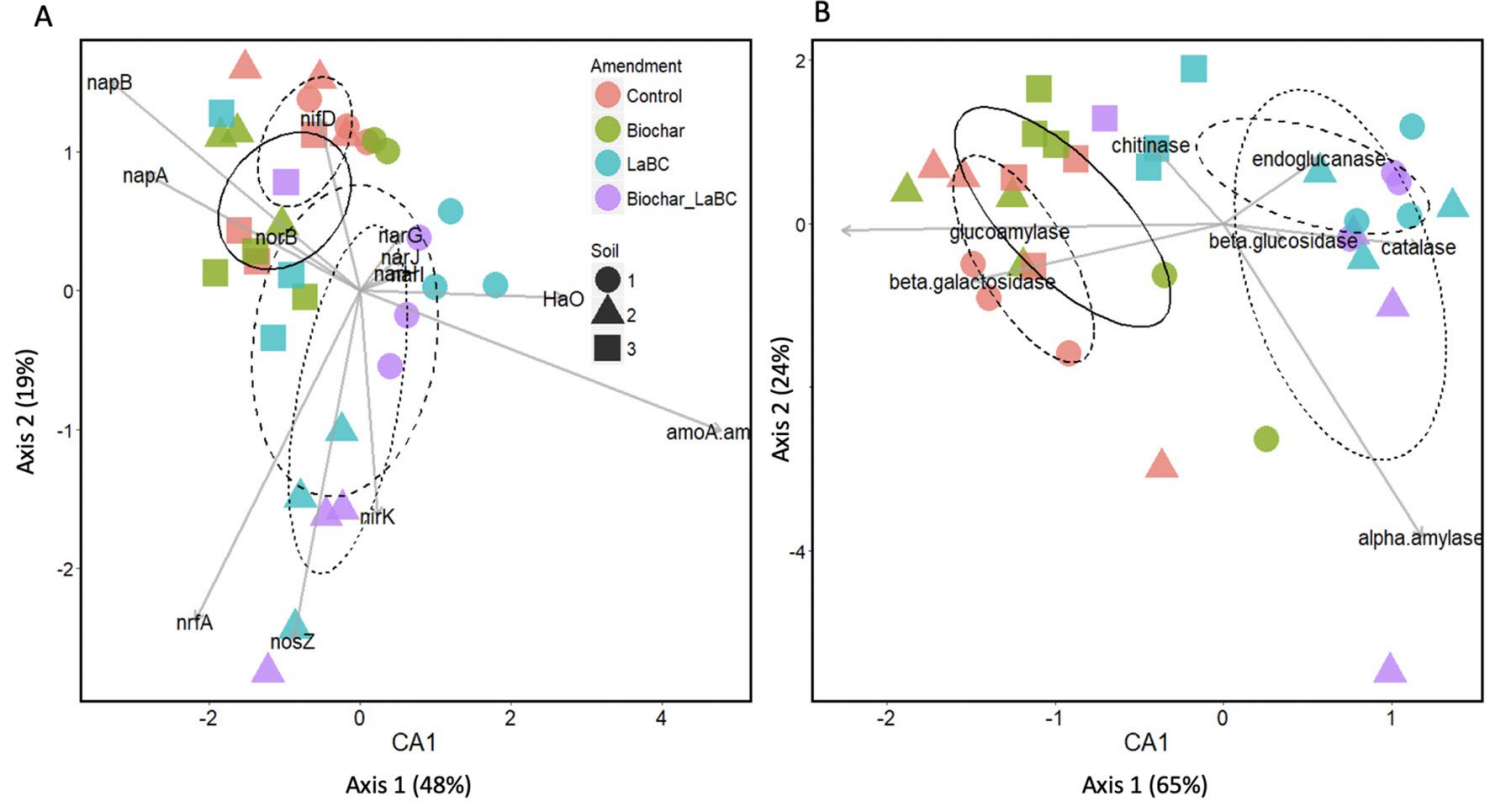
Canonical correspondence analysis (CCA) biplot showing the relationship between predicted gene count (PICRUSt) of N (**A**) and C cycling genes (**B**) and soil amendments for three soils of contrasting textures (Soils 1, 2, 3; sand, sandy loam and clay respectively) amended with LaBC, Biochar and Biochar + LaBC after 8 weeks with subterranean clover. The following C cycling genes that encode for specific enzymes included: *amyA* (α-amylase), *SusB* (glucoamylase), *LacZ* (β-galactosidase), *bglx* (β-glucosidase), *SrpA* (catalase), *ChiC* (chitinase) and *bcsZ* (endoglucanase). The following N cycling genes that encode for specific enzymes included: nitrogen fixing gene *nifD* (nitrogenase); nitrifying genes *amoA/amoB* (ammonia monooxygenase) and *Hao* (hydroxylamine oxidoreductase) and denitrifying genes *norB* (nitric oxide reductase), *narG* (nitrate reductase), *nirK* (nitrite reductase), *nosZ* (nitrous oxide) and *nrfA* (nitrite reductase–ammonium forming). Arrows represent the measured variables (pH, NH_4_^+^, MBN). The ellipses show how the samples separate according to treatment.

Relationships between soil amendments and N and C cycling genes were investigated using CCA analysis (Fig. 4). For Soils 1 and 2, the abundance of functional N genes separated according to treatment along axis 1 (Fig. 4A). The control and biochar treatments clustered with increasing abundance of genes involved in nitrate reduction (*napA*, *napB*), nitric oxide reduction (*norB*) and N fixation (*nifD*). The LaBC and LaBC + biochar samples separated further along axis 2 in relation to soil type. For Soil 1, the LaBC and LaBC + Biochar treatments were associated with an increase in abundance of nitrifying genes (*HaO* and *amoA*). In contrast, the LaBC and LaBC + Biochar treatments were associated with an increase in abundance of denitrifying genes (*nirK*, *nrfA* and *nosZ*). A similar trend was observed for C degrading genes (Fig. 4B) with the control and biochar treatments separating from LaBC and LaBC + Biochar along the axis 1. For C cycling genes, the soil effect was less pronounced than for N cycling genes. Predicted starch and hemicellulose degrading genes *LacZ* and *SusB* (encoding for β-galactosidase and glucoamylase) were higher in abundance in the control soil and soil amended with biochar. Soil from the LaBC and LaBC + Biochar treatments were both associated with a higher abundance of genes associated with degradation of cellulose (*bglx* and *bcsZ* encoding for β-glucosidase and endoglucanase, respectively), chitin (*ChiC* encoding for chitinase) and lignin (*SrpA* encoding for catalase).

**Figure 5:**
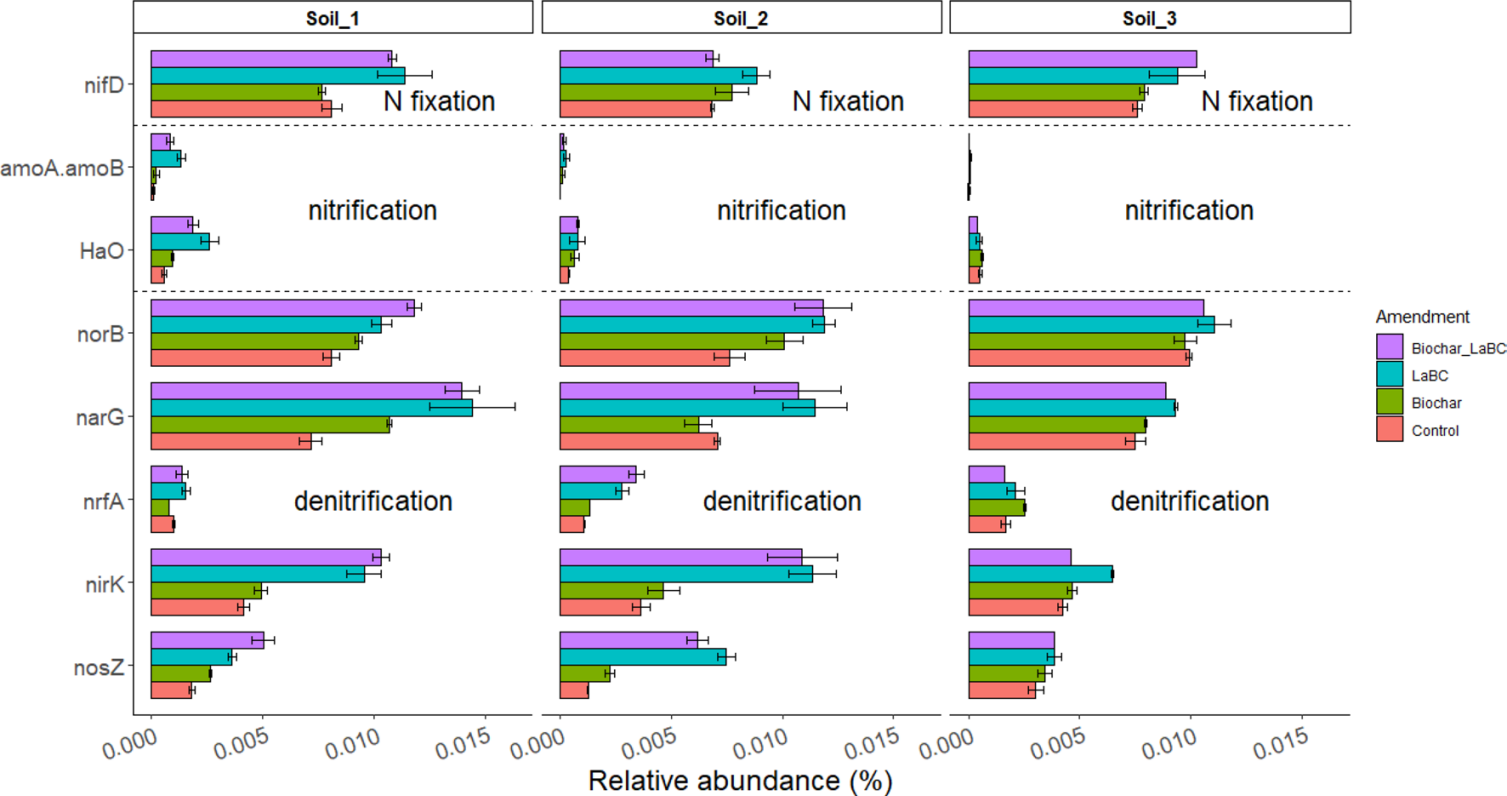
Predicted N gene count (PICRUSt) for the most abundant N cycling genes detected in three soils of contrasting textures (Soils 1, 2 and 3; sand, sandy loam and clay respectively) amended with LaBC, Biochar and Biochar + LaBC after 8 weeks with subterranean clover. Bars represent the mean of each treatment and error bars are the standard error of the mean (n = 3).

Soil type and type of soil amendment both influenced the in silico predicted relative abundance of N cycle genes (Fig. 5). Application of LaBC with or without biochar increased the relative abundance of N-cycling genes associated with N_2_ fixation, nitrification and denitrification (Fig. 5) for Soils 1 and 2. There was a similar effect for Soil 3 but only for soil amended with LaBC or biochar alone. When biochar was combined with LaBC, there was a marked reduction in abundance of genes associated with N_2_ fixation, nitrification and denitrification (Fig. 5).

## Discussion

This research investigated microbial processes underpinning interactions between the biosolids product LaBC, which contained clay and lime, and a woody biochar to predict conditions under which beneficial interactions might occur. Biosolids are known to be a source of nutrients and micronutrients, as well as a contributor of organic matter for improved soil structure, quality and biological activity^3,13,44,45^. However, there is also the possibility of N leaching and contamination^12,21^, especially when they are applied to coarse-textured soils^46^. While co-application of biosolids and biochar can mitigate nitrate leaching^17^ associated with physical and chemical properties of the biochar, changes to N cycling pathways in the soil could also contribute to loss of N. Therefore, our investigation focused on microbial processes associated with co-application of LaBC and biochar in soils of different texture in relation to N-use efficiency. It was expected that the clay^23^ and lime^24^ in LaBC^20^ would also influence N cycling in these soils.

As hypothesised, application of LaBC increased subterranean clover shoot biomass in the two course-textured soils as well as the clayey soil, and this occurred in parallel with increases in soil pH. All three soils were acidic and N deficient and the lime content of LaBC would have improved conditions for growth of subterranean clover^20,47^. However, when LaBC was co-applied with biochar, there was no additional plant growth benefit in any of the soils. Although biochars differ considerably^34^, some forms have more beneficial effects on plant growth in longer-term studies^48^. Our study aligns with previous observations that the same wood-based biochar was ineffective in increasing plant growth in another coarse textured soil^17,49^.

Second, we hypothesised that co-application of LaBC and biochar would reduce the potential for nitrate leaching in the two coarse textured soils. Previously, co-application of biosolids with biochar were shown to reduce nitrate leaching in two non-sandy pasture soils in New Zealand^14,17^. In our study, co-application of LaBC and the wood-based biochar did reduce the potential for N leaching following soil amendment with LaBC in the two coarse textured soils, based on lower soil nitrate. For the two New Zealand pasture soils, a mechanism for reduced nitrate leaching associated with co-application of biochar and biosolids was not identified^17^ although a possible mechanism is that some biochars have both polar and non-polar sites on their surfaces capable of adsorbing a number of inorganic N compounds^14,27,28^. The extent of adsorption varies among biochars^50,51^ with some studies even reporting that mineral N was unaffected by biochar addition^52,53^. Another mechanism by which biochars may reduce mineral N loss from soil involves high surface charge density that improves N retention via increased cation and anion exchange capacity^27,30^. This is a plausible mechanism in our coarse-textured soils (Soils 1 and 2) which are likely to have a low cation exchange capacity^54^. Overall, co-application of LaBC and biochar reduced net mineral N in soil compared to LaBC applied alone to the two course-textured soils. Hence, there is a possibility that some N released from the biosolids in LaBC was adsorbed when co-applied with biochar resulting in less leachable soil N^14^. In addition to these mechanisms, biochars may also directly alter activities of the soil microbial community. Changes to bacterial mediated nitrification and denitrification processes and soil nitrate concentration following biochar amendment have been reported^32,33^ but not for co-application of biochar and biosolids. Therefore, our investigation of soil bacteria associated with co-application of LaBC and biochar examined biological mechanisms that may reduce the potential of nitrate leaching.

Land application of biosolids has previously been shown to alter soil microbial community structure^55–57^. In our study, the impact of amending soil with LaBC was largely due to LaBC-induced changes in soil pH and was dependent upon the soil type. This is consistent with previous studies showing soil pH is a key driver of microbial community structure and function^58–62^. Overall, Proteobacteria and Actinobacteria were the dominant phyla in all three soils, jointly accounting for between 40 and 60% of the total bacterial community. Bacteria within these phyla play a major role in soil processes and are commonly reported in natural^63^ and agricultural systems^64,65^. The application of LaBC either alone or in combination with biochar to the two coarse-textured soils led to an increase in the relative abundance of Actinobacteria and Gemmatimonadetes and a decrease in Acidobacteria and TM7. This corresponds with previous studies investigating the impact of biosolids amendment on soil bacterial diversity^55^. Actinobacteria are known for their ability to degrade complex compounds and recalcitrant materials such as starch, cellulose and lignin^59^ that would have been more abundant in the LaBC given its high organic matter content (Table 1). Perhaps this explains why the C-cycling gene data indicated a greater C turnover and availability in LaBC-amended soils, particularly for genes associated with recalcitrant C such as cellulose, lignin and chitin (*bglx*, *bcsZ*, *ChiC* and *SrpA*). Furthermore, members of these phyla, including Glycomycineae, Paeudonocardineae, Streptosporangineae and Propionibacterineae, are particularly associated with biosolids^66^ and may have persisted in the soil following LaBC application^55^. In contrast, little is known of the ecology of Gemmatimonadetes in soil, but recent studies have found this group tends to be more abundant in agricultural soils, especially organically managed soils receiving biosolid amendments^67,68^. Furthermore, this phylum has high desiccation tolerance and is therefore well adapted to drier soils which may explain why they it was higher in relative abundance in the coarse-textured dryland soils used in our study^67–69^.

Among the other major phyla, Proteobacteria and Bacteroidetes were higher in relative abundance in Soil 2 (sandy loam) amended with LaBC compared with other soils and treatments. Proteobacteria represents the largest and the most metabolically and ecologically diverse phylum and is known to include bacteria that are particularly adept at responding to the presence of a variety of C and N compounds in soil^70,71^. Proteobacteria includes metabolic specialist such as N_2_ fixers, denitrifiers, nitrifiers and methanotrophs^70,71^. Firmicutes are also metabolically versatile and are capable of degrading a variety of complex organic materials^72^. As copiotrophic r-strategists, some bacteria within the phyla Bacteroidetes and Proteobacteria have evolved survival strategies such as high growth rates and metabolic versatility to compete for the C resources (particularly labile carbon)^70^. Thus, an increase in labile C supplied by the biosolid amendment could promote the growth of fast-growing copiotrophs within Proteobacteria and Bacteriodetes that thrive in nutrient-rich environments where they outcompete bacteria from slow-growing phyla^70^. In contrast, Acidobacteria and TM7 are described as oligotrophic with lower growth rates and a preference for growing on relatively recalcitrant forms of C^70,73^. This may explain why these phyla occurred at a reduced relative abundance in the LaBC-amended soils^55^. The sensitivity of Acidobacteria and TM7 to addition of LaBC could also be related to the raised soil pH and higher metal ions associated with biosolids^55,74^. Moreover, Acidobacteria are well-known acidophilic oligotrophs with lower growth rates than fasting growing copiotrophs and a preference for growing on relatively recalcitrant forms of C and acid environments^70,73,74^.

Initially, we hypothesed that LaBC would alter the abundance of N-cycling genes in the two coarse-textured soils due to the clay and lime component of LaBC, with no additional effect of adding the biochar. Management practices that involve the addition of lime or clay to sandy soils in south western Australia can ameliorate soil acidity and increase nitrification activity and *amoA* abundance^49,75,76^. LaBC also contains a significant amount inorganic N (Table 1) which could further stimulate nitrification. Although it was not possible to separate potential effects of clay and lime contained within the LaBC from that of the biosolids, it is likely that they had synergistic effects on the microbial community. Further analysis of N cycling gene abundance showed there was a potential increase in both nitrifying and denitrifying genes in the two coarse-textured soils when amended with LaBC alone and in combination with biochar. These responses were not observed in the clayey soil, probably because it was less affected by both the addition of clay and liming effect of LaBC. In contrast, addition of LaBC to the two coarse-textured soils raised their pH by approximately 2 pH units. Interestingly, although application of LaBC had potential to increase N loss via nitrification and denitrification pathways in the two coarse-textured soils based on responses in N cycling functional genes, co-application of biochar with LaBC appeared to mitigate this by reducing soil nitrate without decreasing plant growth. Consequently, LaBC application may have increased the potential for N leaching in the two coarse-textured soils due to higher soil nitrate and N cycling functional gene abundance. However, the elevated potential for N leaching following application of LaBC was likely to have been reduced by co-application of biochar in these soils due to the adsorption capacity of biochar rather than to reduced microbial N cycling.

Finally, we expected that soil microbial biomass would be higher when LaBC and biochar were applied together than when applied separately, especially in the two coarse-textured soils. This was based on structural improvements in the soil microhabitat^42,43^. However, the level of soil microbial biomass N was unchanged after 8 weeks in all soils. In a study using a coarse-textured soil similar to Soil 1, microbial biomass N also remained unaltered following co-application of the same biochar and N fertiliser, and this was attributed mainly to decreased N mineralisation^19^. Interestingly, the higher soil microbial biomass C for the LaBC treatments applied to the two coarse-textured soils indicates potential competition between soil microbes and subterranean clover for N uptake associated with N immobilisation^77,78^. This provides a further mechanism for greater N retention and less nitrate leaching in the soils amended with LaBC with or without biochar. The extent to which the lime and clay incorporated into the LaBC have any additional effects requires further investigation.

In this study, considerable plant available N was released from LaBC when applied to three pasture soils differing in texture. However, addition of the woody biochar to soils in combination with LaBC significantly reduced soil nitrate. The reduction in soil nitrate occurred despite significant increases in predicted relative abundance of genes associated with both nitrification and denitrification, while co-application of biochar had little effect on subterranean clover growth or plant N uptake in any of the soils. The relative abundance of the two dominant phyla, Actinobacteria and Proteobacteria, increased in the coarse-textured soils with LaBC addition. Co-application of LaBC and the woody biochar used here may be more effective if higher application rates of LaBC are used where there is an increased risk of N leaching.

## Methods

### Experimental design

The glasshouse experiment was conducted to assess the efficiency of adding a combination of LaBC and a wood-based biochar to field soils differing in fertility. The experimental design consisted of two factors: soil amendments (4 treatments) and soil (from 3 locations) in a completely randomised block with three replicates (n = 3). The textures of the three soils (Soils 1, 2 and 3) were a sand, a sandy loam and a clayey soil respectively. The soil amendments were (i) LaBC; (ii) Biochar; (iii) LaBC + Biochar, and (iv) an unamended control. Soil and plant samples were collected 8 weeks after sowing.

Plastic pots (115 mm in diameter, 1 L) were lined with polythene bags and filled with 1 kg of soil packed to the respective bulk density of each soil. Dry amendments were added to soil according to treatments and mixed through the soil in each pot prior to sowing. Deionised water was added to each pot to establish 60% field capacity and maintained throughout the experiment. The soil was conditioned for 7 days to accommodate temporal changes in microbial community structure and increased biological activity that occurs when soil is disturbed during sample collection and preparation^79^. Four germinated subterranean clover (*Trifolium subterraneum* L*.*) seeds were sown per pot and thinned to two per pot after seedling emergence. Pots were harvested 8 weeks after sowing.

### Soils and amendments

The soils were collected from within the Chittering Shire (33° 21′ South, 116° 09′ East), north of Perth, Western Australia. They had different crop and fertiliser histories and varied in texture and chemical properties. Soil 1 was from an alternative crop trial at Chittering Landcare Land Use Demonstration Project site with a pasture of mixed grasses and legumes. Soil 2 was from an agroforestry area at the same site with underlying grass pasture. Soil 3 was from a grass pasture, 3 km to the east. Soils were collected from the surface 0–10 cm, air-dried and passed through a 4 mm sieve. The basic soil characteristics analysed using standard laboratory methods^80^ for Soil 1, 2 and 3 respectively were as follows: ammonium N (7.0, 7.0, 8.0 mg kg^−1^); nitrate N (1.0, ≤ 1.0, 13.0 mg kg^−1^); phosphorus (Colwell) (75, 33, 19 mg kg^−1^); potassium (Colwell) (46, 33, 92 mg kg^−1^); sulphur (4.9, 7.1, 6.0 mg kg ^−1^); organic carbon (1.7, 1.4, 4.2%); EC (3.3, 5.2, 6.5 mS cm^−1^); pH (CaCl_2_) (4.3, 4.5, 4.8); pH (H_2_O) (5.2, 5.5, 5.8); bulk density (1.2, 1.4, 1.0 g mL^−1^); sand (90, 73, 25%); silt (3, 10, 6%); clay (7, 17, 69%) and textural classification (sand, sandy loam, clay).

The soil amendments (LaBC and biochar) were dried separately at 40 °C, homogenised using a mechanical crusher, and the 2–4 mm fraction was retained for use. They had a pH values (CaCl_2_) of 8.2 and 7.6 respectively. The biochar used in this experiment was supplied by Simcoa Ltd (Bunbury, Western Australia) and had been produced in 2008 from jarrah wood (*Eucalyptus marginata* Sm) by pyrolysing at a temperature of 550–650 °C for 24 h. The biochar was mixed thoroughly through soil at a rate equivalent to 20 t ha^−1^ (2% (v/v)). This was based on previous studies of biochar in the same soils^43^. LaBC was produced by the Water Corporation of Western Australia^20^ and mixed thoroughly to soil at a rate of 50 t ha^−1^ (wet weight equivalent).

### Harvest and plant analysis

Plants were harvested 8 weeks after sowing. Shoots were cut at ground level and roots were retrieved from soil, washed with de-ionised water to remove any adhering soil particles, dried at 40 °C and weighed. Shoot N concentrations were determined by combustion in a CNS macro elemental analyser (Elementar, vario Macro CNS, Germany). Plant N uptake (mg N plant^−1^) was calculated by multiplying shoot dry biomass by shoot N concentration.

### Soil chemical and biochemical analyses

At harvest, sub-samples of soil (approximately 50 g) were collected after removing roots and homogenising the soil from each pot. Soil pH (1:5 v/v ratio of soil/0.01 M CaCl_2_ suspension) was measured after shaking for 1 h continuously in a mechanical end-over-end shaker^80^. Soil inorganic N (NO_3_^−^–N and NH_4_^+^–N) was determined using fresh soil (10 g dry weight equivalent) extracts of 0.5 M K_2_SO_4_ (40 mL) after shaking in a mechanical end-over-end shaker for 1 h^20^. Microbial biomass N (MBN) and microbial biomass C (MBC) were determined using the chloroform fumigation-extraction method as described previously^81^. Additional sub-samples of soil were retained and stored at − 20 °C for DNA analysis.

### DNA extraction, PCR amplification, sequencing and bioinformatics

DNA was extracted using the MoBio Powersoil DNA isolation kit (Geneworks, Australia) following the manufacturer’s instruction. The DNA extracts were quantified using a Qubit (Qubit; Thermo Fisher Scientific, Australia) and stored at − 20 °C prior to further analysis. The V4–V5 region of the 16S rRNA gene was PCR amplified using 515F and 806 R primers^82,83^, modified with Golay barcodes^84^ fused to Ion Torrent adapters as detailed in Mickan et al.^85^. and library preparation for sequencing on the Ion Torrent PGM (Life Technologies) as detailed in Mickan et al.^85^. Prior to sequencing, the amplicons were gel purified, quantified and adjusted to 10 ng/μL using molecular grade water. Pooled amplicons were further purified using AmPure XP beads (Beckman Coulter, Australia). Templated beads were created and enriched using the Ion PGM Template reaction kit (Life Technologies, USA) followed by the Ion One Touch ES System (Life Technologies, USA) then loaded onto Ion 316 chips. Sequencing was performed on the Ion Torrent Personal Genome Machine (Life Technologies, USA) using the Ion PGM Sequencing 400 kit.

After sequencing, individual sequence reads were filtered within the PGM software to remove the PGM 3′ adaptor as well as low quality and polyclonal sequences. The resulting PGM quality-filtered sequences were exported as FastQ file and split into *.fasta and *.qual files before analysis using the Mothur Pipeline Mothur Version 1.35.1^86^ as detailed in Mickan et al.^85^. Briefly, the sequence data were subjected to quality control and removed if: it was an singleton, an average quality score (Q) was ≥ 20, no ambiguous bases count, no primer mismatches, homopolymers (length ≤ 12) and minimum length 400. The retained sequencing were clustered and unique sequences were aligned against the Silva 106 database and OTU picking was performed at a sequence similarity of 97%^87^. All chimeric sequences were identified using uchime^88^ and removed. The sequence data were finally subsampled to 4500 per sample to ensure fair comparisons between samples.

To investigate the impact of treatment on microbial functional, specifically genes involved in N cycling, Phylogenetic Investigation of Communities by Reconstruction of Unobserved States PICRUSt^82^ was used as described by Mickan et al.^85^. Briefly, OTU picking was done at a sequence similarity of 97% and aligned against the 13.5 Greengenes database within Mothur. Then PICRUSt was performed following the instructions provided by the developers. Finally, the Nearest Sequenced Taxon Index was performed to test the accuracy of the metagenomic predictions. N and C cycling genes, enzymes and pathways were inferred using the KEGG numbers in the metagenome predicted output file. The following C cycling genes that encode for specific enzymes were included in the analysis: *amyA* (α-amylase), *SusB* (glucoamylase), *LacZ* (β-galactosidase), *bglx* (β-glucosidase), *SrpA* (catalase), *ChiC* (chitinase) and *bcsZ* (endoglucanase). The following N cycling genes that encode for specific enzymes were included in the analysis: nitrogen fixing gene *nifD* (nitrogenase); nitrifying genes *amoA/amoB* (ammonia monooxygenase) and *Hao* (hydroxylamine oxidoreductase) and denitrifying genes *norB* (nitric oxide reductase), *narG* (nitrate reductase), *nirK* (nitrite reductase), *nosZ* (nitrous oxide) and *nrfA* (nitrite reductase–ammonium forming).

### Statistical analyses

The experiment was set up as a complete randomised block design with three replicates. Statistical analyses were conducted using Genstat Version 12. Analysis of variance (ANOVA) was used to compare the treatment effects on soil properties and plant growth parameters and the least significant differences (LSD) were determined at P ≤ 0.05. Canonical correspondence analysis (CCA) was used to identify key phyla and N and C functional genes of the bacteria community that were associated with soil treatment, soil type and environmental variables. CCA modelling was conducted using the Vegan Package in R Statistics^89^.

## Data Availability

The datasets generated during and analysed during the current study are available from the corresponding author on reasonable request.
